# RPS3 Promotes the Metastasis and Cisplatin Resistance of Adenoid Cystic Carcinoma

**DOI:** 10.3389/fonc.2022.804439

**Published:** 2022-06-30

**Authors:** Xi Rui, Zixian Huang, Rui Chen, Yongju Chen, Yan Wang, Zhiquan Huang

**Affiliations:** ^1^Department of Oral and Maxillofacial Surgery, Sun Yat-sen Memorial Hospital, Sun Yat-sen University, Guangzhou, China; ^2^Guangdong Provincial Key Laboratory of Malignant Tumor Epigenetics and Gene Regulation, Sun Yat-sen Memorial Hospital, Sun Yat-sen University, Guangzhou, China

**Keywords:** ribosomal protein s3, adenoid cystic carcinoma, metastasis, invasion, cisplatin resistance

## Abstract

**Background:**

Adenoid cystic carcinoma (ACC) is a malignant tumor in salivary gland tissue, that is characterized by strong invasiveness and lung metastasis, leading to poor survival rates. RPS3 is been reported to be associated with the biological functions of tumor cells. This study explored the regulatory effect of RPS3 in ACC to provide new therapeutic targets for ACC therapy.

**Methods:**

We reviewed the clinical and pathologic data of 73 ACC patients. The expression of RPS3 was examined in ACC by immunohistochemistry. Transwell, wound healing, half-maximal inhibitory concentration (IC50) and other experiments were used to determine the regulatory effect of RPS3 on ACC functions. Coimmunoprecipitation and mass spectrometry analysis were used to detect the binding proteins of RPS3, mechanisms by which RPS3/STAT1/NF-kB signaling regulates ACC behavior were assessed using western blotting (WB), qPCR, etc. To explore the regulatory effect of RPS3 on ACC *in vivo*, we constructed nude mouse sciatic nerve infiltration model and a lung metastasis model for studies.

**Results:**

High RPS3 expression was associated with metastasis and a poor prognosis in ACC patients. Inhibition of RPS3 expression reduced ACC migration, invasion and cisplatin resistance, and overexpression of RPS3 promoted ACC migration, invasion and cisplatin resistance. Further experiments revealed that RPS3 can activate the STAT1/NF-kB signaling pathway and regulate ACC behavior through binding to STAT1. The incidence of sciatic nerve infiltration and lung metastasis in nude mice after RPS3 knockdown was lower than that of the control group *in vivo*.

**Conclusion:**

RPS3 is highly expressed and associated with the prognosis and survival of ACC patients. The RPS3/STAT1/NF-kB pathway may play an important regulatory role in ACC migration, invasion and chemoresistance. As a new therapeutic target of ACC, its clinical application value is worthy of attention and further exploration.

## Introduction

Adenoid cystic carcinomas account for approximately 1% of head and neck malignancies and are the most common malignancy of the salivary glands (1, 2). ACC mostly occurs in salivary gland tissue and is characterized by strong invasiveness, frequent hematogenous metastasis, and rare lymph node metastasis. The prognosis of ACC is poor, and advances in treatment have had no significant effect on ACC treatment outcomes. The low long-term survival of patients is closely associated with relapse and metastasis.

Treatment of ACC is usually radical surgical resection and postoperative radiotherapy (3, 4). Chemotherapy is generally used when patients show rapid progression or severe symptoms (5). Cisplatin is the first-line drug for postoperative chemotherapy in oral cancer patients (6), but tumor resistance to cisplatin chemotherapy is increasingly serious (7, 8). How to effectively improve the sensitivity of postoperative chemotherapy is a hot and difficult problem in oral cancer research. Furthermore, targeted molecular therapy in ACC patients has been extensively studied, but the slow course of the disease limits its clinical efficacy. Currently, there is an urgent need for research on new treatments to improve therapeutic efficacy in ACC patients.

Recently, some ribosomal proteins (RPs) have been shown to be upregulated in several cancers and to promote tumorigenesis (9, 10). Ribosomal protein S3 (RPS3), a component of the 40S ribosomal subunit, is involved in its initiation and translation (11). In addition to ribosomal function, RPS3 is involved in DNA repair, gene transcription and apoptosis, and tumorigenesis (12, 13). Moreover, RPS3 has been reported to be associated with the radioresistance, chemoresistance and invasive metastasis of tumor cells (14), but its role in the development of ACC remains poorly understood. Therefore, we conducted this study to explore the regulation of ACC cell function by RPS3 to provide new targets for the treatment of ACC.

## Materials and Methods

### Patient Enrollment and Tissue Collection

This study was approved by the Ethics Committee of Sun Yat-sen Memorial Hospital, Sun Yat-sen University. We collected ACC patients admitted to the Oral and Maxillofacial Surgery Department of Sun Yat-sen Memorial Hospital from September 2015 to September 2018. All the patients were diagnosed with ACC in head and neck regions *via* postoperative pathology. No patients were diagnosed with other oral malignancies. Clinical stage was analyzed according to the American Joint Committee on Cancer (AJCC) Staging Manual, 8e. Lymph node metastasis was evaluated according to routine postoperative pathology and immunohistochemistry (IHC) results, and distant metastasis was evaluated based on follow-up and imaging data.

### IHC

**Figure 1:**
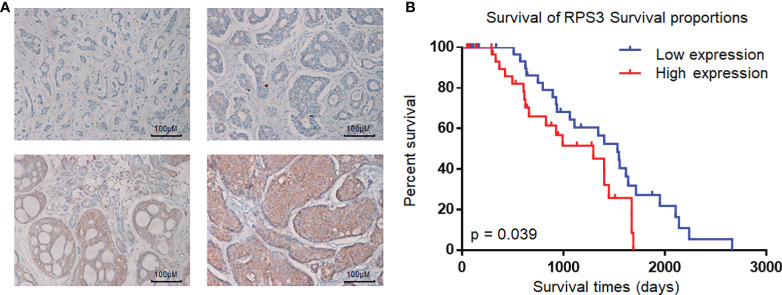
Expression of RPS3 in ACC and analysis of pathological sections. **(A)** IHC scoring criteria: the staining intensity scores were 0 (negative), 1 (weak), 2 (medium), and 3 (strong). **(B)** Kaplan-Meier estimates of the overall survival of ACC patients based on RPS3 expression and survival rates.

Immunohistochemical staining was performed according to the standard protocol. Samples were deparaffinized in xylene and then rehydrated in serial ethanol solutions and distilled water, and antigen repair was performed in a water bath at 95°C with 10 mM sodium citrate buffer (pH 8.0). The samples were cooled in the buffer for 30 min, and then the tissues were incubated with 3% H2O2 for 10 minutes to inhibit endogenous peroxidase activity. The samples were blocked with 5% normal goat serum for 1 h at room temperature, and then the pathological sections were incubated overnight with a primary antibody against RPS3 (#11990–1-AP, ProteinTech, USA) at 4°C. The next day, the biotinylated secondary antibody bound to the streptavidin-HRP complex was added for incubation, followed by 3–3′-diaminobenzidine and hematoxylin and eosin staining. The dehydration and installation processes were performed according to the instructions. IHC staining was assessed by 2 pathologists separately according to the number of positive cells and staining intensity, when there was a conflict between two pathologists, the third pathologist scored. The staining intensity scores were 0 (negative), 1 (weak), 2 (medium), and 3 (strong) (**Figure 1A**), and the percentages of positive cell scores were 0 (0%), 1 (1–25%), 2 (26–50%), and 3 (≥ 50%), respectively. The total score was obtained by adding the staining intensity score and the percentage score. An overall score greater than or equal to 3 was considered to indicate high expression.

### Cell Transfection

For transient transfection using small interfering RNAs (siRNAs), siRNAs targeting RPS3 were synthesized by IGE Biotechnology (Guangzhou, China). Transfection was performed with Lipofectamine RNAiMAX reagent (Invitrogen, USA) according to the manufacturer’s protocol. The siRNA sequences are shown in **Table 1**.

**Table 1:** Small interfering RNA (siRNA) and miRNA-mimic used in transfection.

Name	Sense(5’-3’)	Antisense(5’-3’)
siRPS3-1	GGUUGUGGUGUCUGGGAAATT	UUUCCCAGACACCACAACCTT
siRPS3-2	GCAUCAAGGUGAAGAUCAUTT	AUGAUCUUCACCUUGAUGCTT
siSTAT1-1	CUGACUUCCAUGCGGUUGA	UCAACCGCAUGGAAGUCAG
siSTAT1-2	CGGCUGAAUUUCGGCACCU	AGGUGCCGAAAUUCAGCCG
siRNA-NC	UUCUCCGAACGUGUCACGUTT	ACGUGACACGUUCGGAGAATT

For stable expression, lentiviral plasmids harbouring the desired gene were first transfected into 293T cells together with the packaging plasmids pSPAX2 and pMD2.G at a ratio of 5:3:2. HEK293 cells were plated into a 10-cm plate and cultured as previously described. After reaching 70–80% confluence, the cells were transfected with 6 µg of psPAX2, 3 µg of pMD2.G and 10 µg of transfer vector using Lipofectamine 3000 reagent. Forty-eight hours after transfection, the supernatants of each group were collected and used to infect ACC cells for another 48 h. Puromycin-tolerant ACC cells were selected.

### mRNA Expression Analysis

The sequences of PCR primers are shown in **Table 2**; GAPDH was used as an internal reference. Total RNA was extracted according to the manufacturer’s instructions with PrimeScript™ RT Master Mix (Takara, Japan) and reverse transcribed to cDNA in an ABI 9700 Real-Time PCR instrument (ABI, USA). 1 μl cDNA was mixed with the TB Green^®^ Premix Ex Taq™ II (Takara, Japan) in a 20 μl reaction volume; relative mRNA expression was detected with a LightCycler 480 II Real-time PCR instrument (Roche, Swiss). Statistical software was applied for statistical analysis. The reaction conditions were as follows: 94°C for 2 min, 94°C for 20 s, 58°C for 20 s, and 72°C for 20 s for forty cycles. All the reactions were performed in triplicate.

**Table 2:** Information of primers for qRT-PCR and PCR.

Name	Forward primer	Reverse primer
RPS3	CTGGAGTTGAGGTGCGAGTTA	ACAGCAGTCAGTTCCCGAATC
STAT1	ATCAGGCTCAGTCGGGGAATA	TGGTCTCGTGTTCTCTGTTCT
GAPDH	GAGTCAACGGATTTGGTCGT	GACAAGCTTCCCGTTCTCAG

### Western Blot Assay

For protein extraction, the cells were harvested by scraping and then lysed in lysis buffer (#01408, Beyotime, China) containing 1% protease inhibitor cocktail (#CW2200S, CWBIO, China). Following centrifugation at 13000 rpm/min at 4°C for 30 min, the supernatant was collected, and the protein concentration was determined using the BCA Protein Assay Kit (#CW0014S, CWBIO, China). The loading buffer (#CW0027S, CWBIO, China) was diluted and mixed, and the protein was denatured at 95°C for 5 min. An SDS-PAGE gel was prepared according to the instructions of a Beyotime polyacrylamide gel kit. After electrophoresis (90 V, 1.3 h), the proteins were transferred to a PVDF membrane (#P0021S, Beyotime, China) (250 mA, 70 min). The PVDF membrane was incubated with TRIS-buffered saline containing 5% skim milk with 0.1% Tween-20 for 1 h at room temperature for protein blocking. The primary antibody (RPS3 (#11990–1-AP, Proteintech, USA), GAPDH (#60004-1-Ig, Proteintech, USA), STAT1 (10144-2-AP, Proteintech, USA), P-STAT1 (#9167, Cell signaling technology, USA), P65 (#66535-1-Ig, Proteintech, USA), P-P65 (#3033, Cell signaling technology, USA)) was incubated for 16 h in a 4°C shaker. The membrane was washed with Tris-buffered solution containing 0.1% Tween-20, and the secondary antibodies [goat anti-mouse IgG-HRP (#sc-2005, Santa Cruz Biotechnology, USA) and goat anti-rabbit IgG-HRP (#sc-2004, Santa Cruz Biotechnology, USA)] were incubated for 1 h at room temperature. Chemiluminescence, photography and gel image analysis were carried out according to standard protocols.

### Half-Maximal Inhibitory Concentration

Cisplatin resistance was determined by using a cell-based half-maximal inhibitory concentration (IC50) method. The cells were seeded at 10,000 cells/well into 96-well plates. Cisplatin was added at concentrations of 0 µM, 2.5 µM, 5 µM, 10 µM, 20 µM, 40 µM, 80 µM, and 160 μM to each group for 48 h. Cell relative absorbance was measured by the Cell Counting Kit-8 (CCK-8) method.

### Scratch-Healing Assay

When the cell density in 6-well plates reached 90% -100%, cells were evenly scraped with small pipette tips, washed three times with PBS, and then supplied with fresh medium containing 1% FBS to continue culture. Photographs were taken by microscopy at 0 h, 12 h, 24 h and 48 h. The mean scratch healing width and scratch healing rate were calculated for each group (× 100, Nikon 80i, Nikon Corporation).

### Transwell Assays

A total of 30,000 cells in medium (150 μL containing 1% FBS) was added to the upper chamber (8-μm diameter [#3422, Corning)]. For the Transwell migration experiments, the upper compartment did not contain the addition of matrix; however, matrix was added to the upper compartment for the Transwell invasion experiments. Then, 700 μL of complete DMEM containing 10% FBS was added to the lower 24-well plate. After 24 h, the cells that did not pass through the membrane were removed with a cotton swab; the remaining cells were stained with 500 μl 4% paraformaldehyde for 20 min and 500 μL 1% crystal violet for 30 min at room temperature, the cells were counted under a microscope (× 100, Nikon 80i, Nikon Corporation).

### Protein Coimmunoprecipitation and Protein Mass Spectrometry Analysis

#### Co-IP

Cells from different groups were plated in 10-cm cell dishes with a cell confluence of approximately 90%. NETN buffer was mixed with a protein phosphatase inhibitor and added to plates to lyse the cells (1 ml cell lysate per plate, 20 min on ice). The protein supernatant was collected by centrifugation at 13,000 rpm/min for 30 min, and 50 µl of each sample was left as a positive control. One microgram of antibody was added to the lysate (4°C rotary incubation for 1 h), and protein G magnetic beads were washed twice with NETN buffer and then added to the lysate (4°C on a rotary shaker overnight). The next day, the beads were washed 5 times with NETN followed by 50 µl NETN + loading buffer at 95°C for 5 min. The supernatant was harvest, and immunoblotting was performed.

#### Mass Spectrometry Analysis

Protein samples cultured overnight in the Co-IP experiment were used for protein analysis. After 100 μl of protein lysate (10 mM DTT/100 mM NH4HCO3 solution), the cells were shaken at 56°C and 1,300 rpm for 30 min, and the supernatant was removed by centrifugation. Then, 100 μl 100 mM iodoacetamide was added, and the supernatant was removed by centrifugation after 30 min. Then, 100 μl NH4HCO3 (100 mM) resuspended beads with 1 µg trypsin and shaken for 13 h (37°C, 1,000 rpm). After terminating digestion with 10% trifluoroacetic acid at 0.4 l (TFP), the protein were concentrated and redissolved for protein profiling. Gene ontology (GO) and Kyoto Encyclopedia of Genes and Genomes (KEGG) pathway analysis of differentially expressed genes was performed.

### NF-kB Activation and Inhibition

TNF-α (#HY-P1860, MCE) and Bay11-7082 (#19542-67-7, MCE) was used as NF-kB pathway activator and inhibitor respectively, were stored at -80°C in 1 mM storage solution. For activation experiments, the final inhibitor concentration was 5 μM, and the cells were treated for 1 h. For inhibition experiments, the final inhibitor concentration was 6 nM, and the cells were treated for 24 h.

### Animal Model Construction

Five-week-old female BALB/C nu/nu mice were provided by Baishitong, Guangzhou, and were reared under SPF conditions. They were randomly divided into 2 groups, (control group and sh-RPS3 group), and each group was randomly divided into the sciatic nerve infiltration group and lung metastasis group. ACC-LM cells were cultured *in vitro* to the exponential growth phase, digested with trypsin, pipetted into a single cell suspension, centrifuged at 1000 rpm/min for 5 min, The supernatant was removed, and PBS solution was added to dilute the cells according to the required concentration, after which the cells were prepared into a single cell suspension (mice in the sciatic nerve infiltration group received: 200 μl/5^106 cells/mice, and mice in the lung metastasis group received: 200μl/1x106 cells/mice).

After anesthetizing nude mice with ether, the cell suspension was injected into the unilateral sciatic nerve of the mice (middle 1/3 of the line connecting the knee to the root of the tail). The mouse weight and tumor volume were observed every 5 days, and the tumor growth curve was calculated and drawn according to the formula Volume=(Lenght x Width^^2^)/2.

A sticker was stuck to the hindlimb on the hindlimb of mice, and the ability of the mice to get out of the sticker restraint and the time it took to move were observed. Dysfunction was manifested by the inability of normal voluntary movement of the limbs and the weakness of hindlimb grasping, while healthy mice could quickly get rid of the restraint. The lungs of mice in the lung metastasis group were dissected 1.5 months after the tail vein injection, and the lung metastasis was observed. The tumor specimens were embedded in paraffin, and the tissue sections were stained with HE and observed under a high-power microscope.

### Statistics

Data analysis was carried out using SPSS 22.0 software (IBM, Chicago, Illinois). In this study, the normal distribution and the homogeneity of variance for the experimental data were analyzed using the Kolmogorov test and expressed as the mean ± standard deviation (M ± SD). Parameters obeying a normal distribution were assessed by independent sample t tests; otherwise, nonparametric tests were used. Univariate survival analysis was calculated using the Kaplan-Meier method. Comparisons of different times and concentrations were performed by repeated measurement ANOVA, and pairwise comparisons between groups were performed using the Bonferroni *post hoc* method. P <0.05 was considered to indicate a statistically significant difference.

## Results

### Clinical Patient Data and Analysis of Pathological Sections

To assess the relationship between the expression level of RPS3 in ACC and the prognosis of patients, a total of 73 patients with ACC (35 males and 38 females), aged 18~70 years (mean age 42 ± 8.5 years), were included in this study; 37 had high RPS3 expression, and 36 had low RPS3 expression. The clinical data of the patients are shown in **Table 3**, and there was no significant difference in general features between the two groups. Similarly, there was no significant difference in tumor site or clinical stage between the two groups (P>0.05). However, the incidence of lymph metastasis (P= 0.014) and pulmonary metastasis (P= 0.016) in patients with high RPS3 expression was higher than that in patients with low RPS3 expression. As shown in **Figure 1B**, Kaplan-Meier estimates of the overall survival of ACC patients showed that patients with high expression of RPS3 tended to have poorer survival rates (P=0.039). Based on clinical patient data and immunohistochemical results of pathological sections, we found that high expression of RPS3 was associated with metastasis and a poor prognosis in ACC patients.

**Table 3:** Association of RPS3 expression with the features of ACC patients.

Variables	Expression of RPS3		P value
	Low expression	High expression	
Age(y)			0.537
≤50	21	18	
>50	16	18	
Gender			0.561
Male	19	16	
Female	18	20	
Site			0.207
Palate	15	9	
Tougue	3	2	
Mouth floor	6	8	
Submandibular	3	6	
Glands	8	6	
Others	2	5	
Clinical stage			0.295
1	4	2	
2	3	11	
3	11	9	
4	19	14	
Lymphatic metastasis			0.014
No	13	23	
Yes	24	13	
Pulmonary metastasis			0.016
No	20	29	
Yes	17	7	

RPS3 High expression 37(50.68%), Low expression 36(49.32%).

### Overexpression of RPS3 Increased ACC Cells Cisplatin Resistance, Migration and Invasion

**Figure 2:**
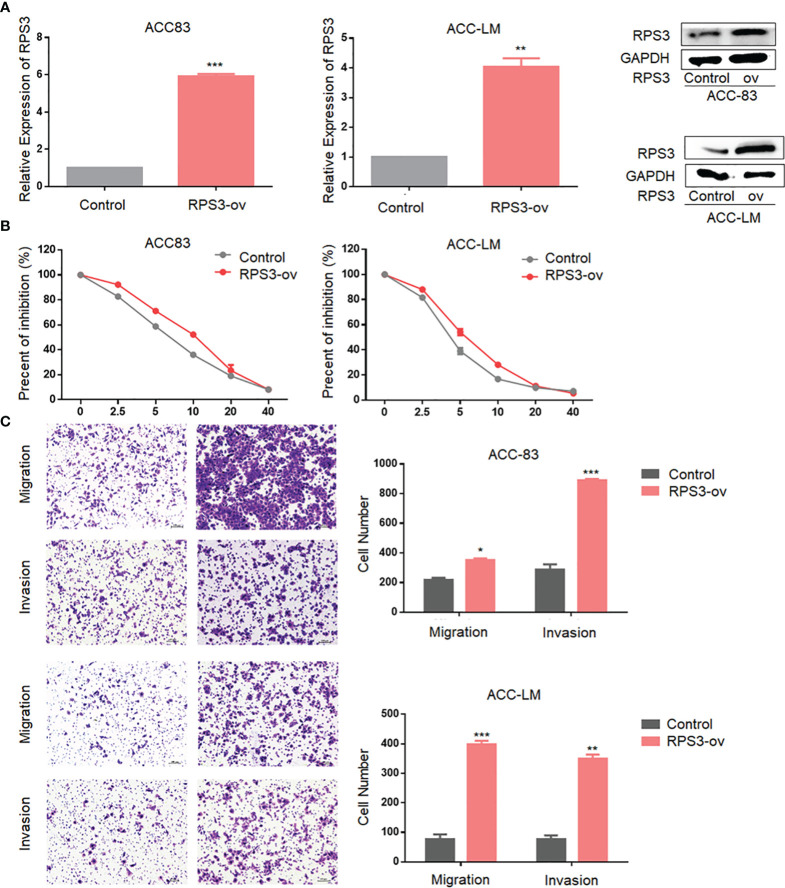
Overexpression of RPS3 increased ACC migration, invasion and cisplatin resistance. **(A)** qPCR and WB experiments showed that the mRNA and protein levels of RPS3 were increased after transfection with plasmids in ACC-83 and ACC-LM cells. **(B) **IC50 experiments showed enhanced cisplatin resistance in ACC-83 and ACC-LM cells after RPS3 overexpression. **(C)** The Transwell assay results showed increased migration and invasion in ACC cell lines after RPS3 overexpression. *P < 0.05; **P < 0.01; ***P < 0.001.

We found that RPS3 was associated with a poor prognosis in ACC. To order to clarify the regulatory effect of RPS3 on the biological function of ACC cells, we conducted functional experiments in ACC cell lines overexpression of RPS3. As shown in **Figure 2A**, we constructed stable ACC-83 and ACC-LM cells overexpressing RPS3 and verified successful overexpression. The results of subsequent IC50 experiments showed enhanced cisplatin resistance in ACC-83 and ACC-LM cells after RPS3 overexpression (**Figure 2B**). After overexpression of RPS3, more ACC cells crossed the membrane to the lower level of the chamber and the results of the Transwell assay showed increased migration and invasion in ACC cell lines, the difference is statistically significant(P<0.05, **Figure 2C**). Therefore we speculated that the cisplatin resistance and invasion ability of ACC cells were improved after overexpression of RPS3.

### Inhibition of RPS3 Expression Reduced ACC Cells Migration, Invasion and Cisplatin Resistance

**Figure 3:**
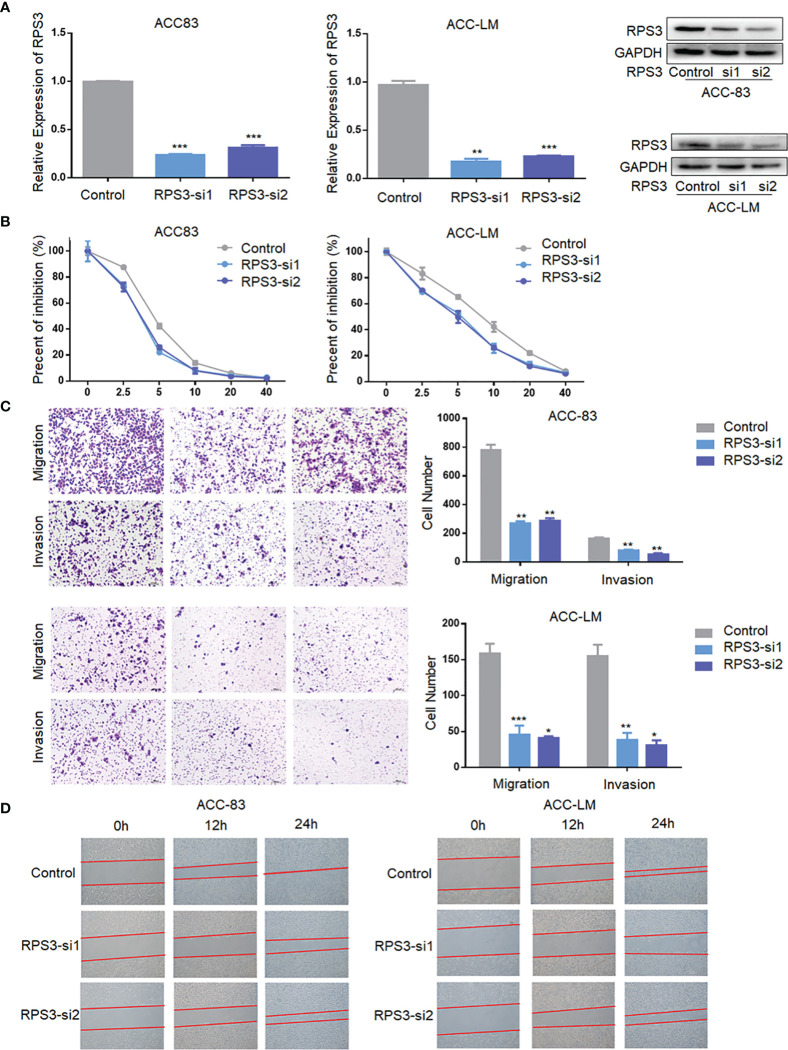
Inhibition of RPS3 expression reduced ACC migration, invasion and cisplatin resistance. **(A)** qPCR and WB experiments showed that the mRNA and protein levels of RPS3 were decreased after transfection with siRNA in ACC-83 and ACC-LM cells. **(B)** The IC50 value showed cisplatin chemoresistance upon downregulation of RPS3. **(C)** ACC cell migration and invasion were inhibited upon downregulation of RPS3, according to the transwell assay. **(D)** ACC cell migration was inhibited upon downregulation of RPS3, according to the wound-healing assay. *P < 0.05; **P < 0.01; ***P < 0.001.

After initially confirming that RPS3 could promote the migration, invasion and cisplatin resistance of ACC, we attempted to suppress the metastatic behavior and cisplatin resistance by knocking down the level of RPS3. We used RNA interference technology to knockdown the expression of RPS3 in ACC-83 and ACC-LM cells, as shown in **Figure 3A**, WB and qPCR were used to verify the knockdown efficiency of the two siRNAs. In terms of cisplatin resistance, **Figure 3B** shows that after knockdown of RPS3, the IC50 of cisplatin in ACC cells decreased. The results from the Transwell assay showed that the migration and invasion abilities of ACC cells decreased after RPS3 was knocked down (**Figure 3C**). The wound-healing assay results in **Figure 3D** also show a decreased migration ability after RPS3 knockdown. Through cell function experiments, we confirmed that inhibiting the expression level of RPS3 would reduce cisplatin resistance and migration, invasion of ACC cells.

### RPS3 Interacted With STAT1 and Participated in the Regulation of ACC

**Figure 4:**
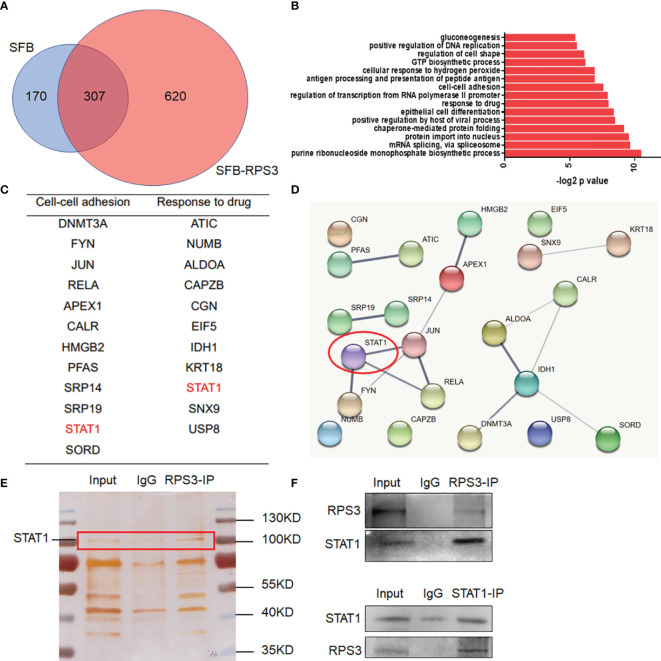
RPS3 interacted with STAT1. **(A)** A total of 620 differentially expressed proteins specifically bound to RPS3. **(B)** GO analysis of the differentially expressed genes. **(C)** Related proteins were enriched among pathways associated with migration, invasion and drug sensitivity. **(D)** The interconnections between related genes. **(E)** Silver staining experiments confirmed the mutual binding of RPS3 and STAT1. **(F)** IP experiments confirmed the mutual binding of RPS3 and STAT1.

After clarifying the functional regulation of RPS3 in ACC, we attempted to identify proteins that interact with RPS3, and screened key factors related to migration, invasion and chemotherapy resistance, in an attempt to explore the regulatory mechanism. We used ACC-83 cells for a Co-IP assay combined with mass spectrometry analysis to find proteins binding to RPS3. A total of 620 differential proteins specifically bound to RPS3 (**Figure 4A**). GO analysis of the differentially expressed genes indicated that the related proteins were enriched among pathways associated with migration, invasion and drug sensitivity are shown in **Figures 4B–D**. Then, we analyzed the relationship between these differentially enriched proteins to identify the common differentially expressed gene—signal transducerand activator of transcription 1 (STAT1). Subsequent silver staining experiments confirmed the mutual binding of RPS3 and STAT1 (**Figure 4E**). In the further study of mechanism, IP experiments confirmed the mutual binding of RPS3 and STAT1 (**Figure 4F**). Based on these above experiments and database analysis, we speculated that RPS3 plays a role in regulating drug resistance and migrating invasion by regulating the directly bound protein STAT1.

### RPS3 Was Involved in the Regulation of the STAT1/NF-kB Signaling Pathway

STAT1 is an important protein connecting cell membrane receptors and effectors in signal transduction, and can undergo phosphorylation in the cytoplasm and polymerize to form homologous or heterologous dimers, after which it can enter the nucleus to promote the transcription of target genes.

**Figure 5:**
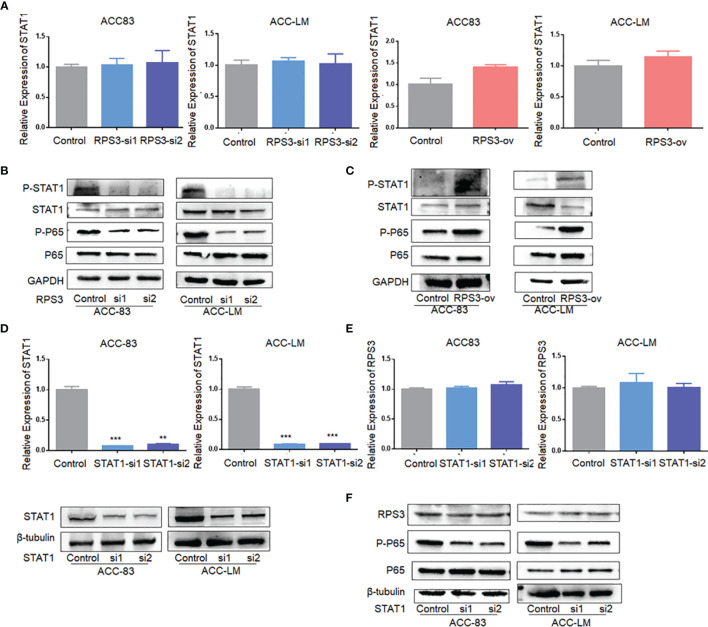
RPS3 was involved in the regulation of the STAT1/NF-kB signaling pathway. **(A)** QPCR showed no significant change in STAT1 mRNA levels in ACC cells after changing the expression RPS3. **(B)** WB showed a significant decrease in STAT1 and P65 phosphorylation after RPS3 expression was reduced. **(C)** WB showed increase activation of STAT1 and P65 after RPS3 overexpression. **(D)** QPCR and WB experiments showed that the mRNA and protein levels of STAT1 were decreased after transfection with siRNA in ACC-83 and ACC-LM. **(E)** QPCR showed there was no change in mRNA levels of RPS3 after STAT1 expression was reduced. **(F)** WB showed that after STAT1 sxpression was redued, there was no change in the posttranscriptional level of RPS3, but P65 activation was reduced. **P < 0.01; ***P < 0.001.

We used WB and qPCR to explore the effect of differential expression of RPS3 on STAT1 activation. There was no significant change in STAT1 mRNA levels in ACC cells after changing the expression RPS3 (**Figure 5A**), while WB showed a significant decrease in STAT1 phosphorylation but not post-transcriptional levels in ACC cells after RPS3 expression was reduced (**Figure 5B**). At the same time, the downstream NF-κB pathway showed a corresponding reduction in activation. WB showed increased activation of STAT1 and P65 after RPS3 overexpression (**Figure 5C**).

Next, we knocked down STAT1 expression in ACC cells, and qPCR and WB showed no significant change in RPS3 at either the mRNA or protein level (**Figure 5D**). Further experiments found that there was no change in pre-transcriptional and post-transcriptional levels of RPS3 after STAT1 knockdown (**Figures 5E, F**). Therefore, in further mechanistic studies, we verified the alterations in NF-kB activation levels after STAT1 knockdown. The WB results showed that knockdown of STAT1 in ACC cell lines similarly reduced P65 activation (**Figure 5F**).

### The RPS3/STAT1/NF-kB Signaling Pathway Regulates the Cisplatin Resistance and Migration-Invasion Behavior in ACC Cells

**Figure 6:**
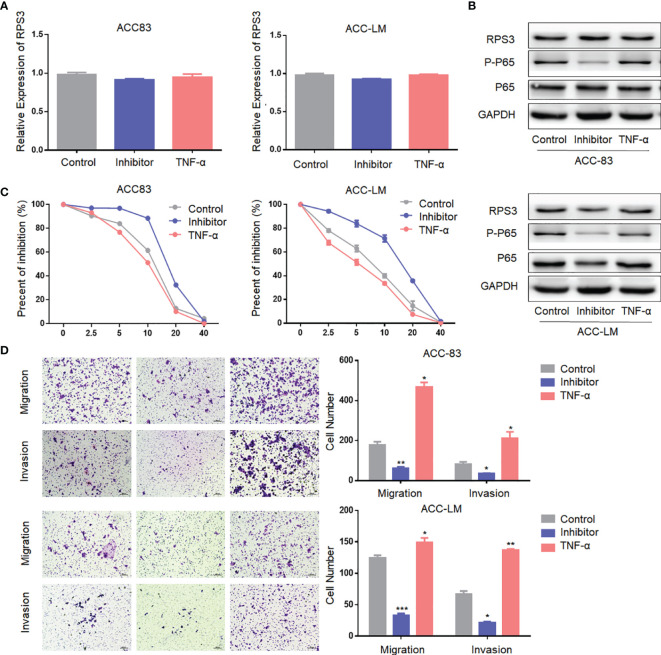
The NF-kB signaling pathway regulate the cisplatin resistance and migration-invasion behavior of ACC cells. **(A)** The use of activators and inhibitors did not alter RPS3 mRNA expression levels. **(B)** The use of activators and inhibitors did not alter RPS3 protein expression levels, but alter the activation of NF-κB signaling pathway. **(C)** Cisplatin resistance decreased in ACC cells after inhibition of the NF-kB pathway, while activation of the NF-kB pathway promoted cisplatin resistance. **(D)** Migration and invasion decreased in ACC cells after inhibition of the NF-kB pathway, while activators promoted migration and invasion. *P < 0.05; **P < 0.01; ***P < 0.001.

To further confirm our hypothesis, we treated ACC cells with activators and inhibitors of NF-κB, and the use of activators and inhibitors did not alter RPS3 expression levels (**Figures 6A, B**). The activation of NF-κB after treatment with activators and inhibitors is shown in **Figure 6B**. The results are consistent with previous findings that cisplatin resistance decreased in ACC cells after inhibition of the NF-kB pathway, while activation of the NF-kB pathway promoted cisplatin resistance (**Figure 6C**). Similarly, migration and invasion decreased in ACC cells after inhibition of the NF-kB pathway, while activators promoted migration and invasion (**Figure 6**). In general, The RPS3/STAT1/NF-kB signaling pathway regulates the cisplatin resistance and migration-invasion behavior of ACC cells.

### *In Vivo* Experiments to Verify the Effect of RPS3 Knockdown on ACC Metastasis in Nude Mice

**Figure 7:**
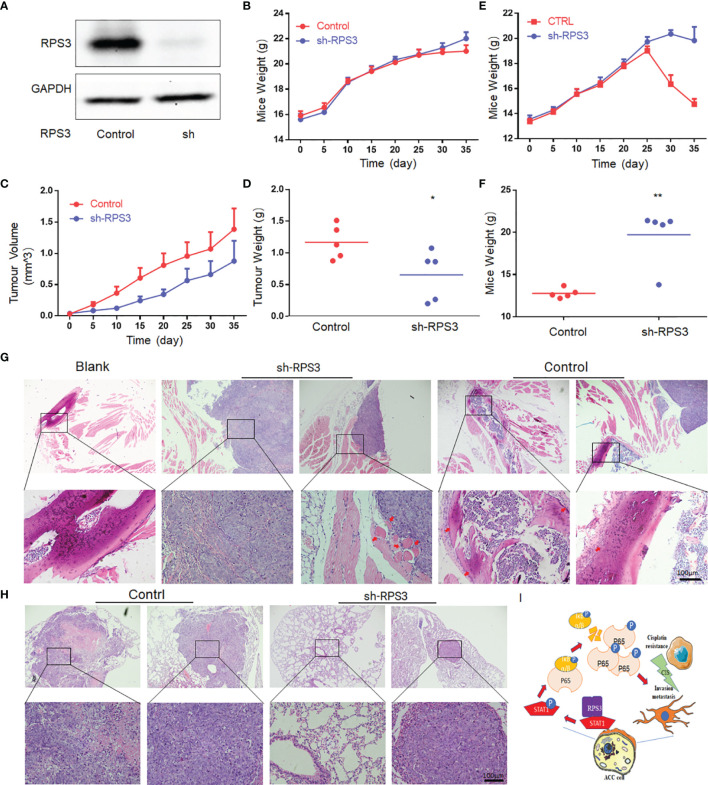
Knockdown of RPS3 inhibited ACC invasion of the sciatic nerve and lung metastasis in nude mouse model. **(A)** Western blot verifies that a stable RPS3 knockout ACC-LM cell line was successfully constructed. **(B)** Weight change curve of in the sciatic nerve invasion nude in the mouse model. **(C)** Tumor growth curve of the sciatic nerve invasion nude mouse model. **(D)** The tumor weight of the mice in the knockdown RPS3 group was lower than that in the control group (P<0.05). **(E)** Weight change curve of nude mouse model of lung metastasis. **(F)** The weight of the mice in the knockdown RPS3 group was significantly higher than that in the control group (P<0.05). **(G)** HE staining was used to observe the tumor and sciatic nerve (red arrows point to nerves. In the RPS3 knockdown group, there was no sciatic nerve around some tumors, and some tumor tissues compressed the nerve but did not surround the nerve. while the tumor in the control group wrapped the compressed nerve, and the nerve was twisted and deformed, and even tumor tissue infiltration was observed in some nerves. **(H)** HE staining showed the tumor tissue of the control group and the sh-RPS3 group; some mice in the sh-RPS3 group did not have tumors. **(I)** Schematic model of the mechanism by which the RPS3/STAT1/NF-kB signaling pathway regulates cisplatin resistance and migration-invasion behavior of ACC cells. * P < 0.05;* * P < 0.01.

The previous experiments confirmed that the RPS3/STAT1/NF-kB signaling pathway can promote ACC migration and invasion, and knockdown of RPS3 can significantly inhibit the migration and invasion ability of ACC *in vitro*. This suggests that inhibition of RPS3 expression may be a key approach to treat ACC metastasis. Therefore, we constructed an ACC-LM cell line that stably knocked out RPS3 (**Figure 7A**), and verified by western blot.

The mouse weight and tumor volume in the sciatic nerve invasion group were recorded every 5 days after tumor formation. The results showed that the weight of mice in both the control and sh-RPS3 groups gradually increased, and there was no significant difference between the two groups (**Figure 7B**). However, the results of the tumor growth curve showed that the tumor volume of the sh-RPS3 group grew significantly slower than that of the control group (**Figure 7C**), and the tumor was removed out at the end of the experiment on the 35th day. The tumor weight of the sh-RPS3 group was significantly lower than that of the control group, and the difference was statistically significant (**Figure 7D**, P<0.05).

The results of the lung metastasis group showed that the weight of the mice in the control group increased gradually and decreased sharply after 25 days, in contrast, the weight of the mice after knocking down RPS3 gradually increased and started to decrease after 1 month, but the decrease was less than that of the control group (**Figure 7E**). On the 40th day, 2 mice in the control group died, therefore, this part of the animal experiment was terminated. At the end of the experiment, the weight of the mice in the sh-RPS3 group was significantly higher than that of the control group (**Figure 7F**), the difference was statistically significant (P<0.05).

The mice in the sciatic nerve invasion group were subjected to a sticker experiment and the movement of the hindlimb on the tumor-bearing side was observed (**Supplementary Videos 1**–**3**). As shown in **Table 4**, with the growth of tumors, 2 mice in the control group were observed to have dyskinesia on the 10th day after tumor formation. The mice were unable to move their hindlimbs freely, had weak grasping and could not escape the stickers. The final rate of dysfunction in this group was 80%. However, knocking down the expression of RPS3 can reduce the occurrence of dysfunction. Only one mouse was found to have dysfunction on the 20th day, and the final incidence rate was 20%. The tumor was dissected and it was observed that the nerves invaded by the tumor were compressed and surrounded. After dissecting and separating the nerves compressed and encapsulated by the tumor, it was observed that they were significantly distorted and compressed by edema compared (**Supplemental Figure 1A**). The tumor tissue was embedded, sectioned, and stained with HE. Through the tissue section, we observed edema and thickening of the invaded epineurium, and the nerves were wrapped and squeezed by tumor cells, tumor tissue was even observed in part of the nerve (**Figure 7G**). We counted the number of mice with neuroinvasion in the 2 groups. **Table 4** shows that compared with the control group, all 5 mouse (100%) showed neuroinvasion, and only 1 mice (20%) displayed neuroinvasion after inhibiting the expression of RPS3.

**Table 4:** ACC invasion of sciatic nerve and hindlimb motor dysfunction after RPS3 knockdown.

	Disfunction (day)								Trial Endpoint		
	0	5	10	15	20	25	30	35	Disfunction Rate (%)	Neuro-invasion	Neuro-invasion (%)
Control	0	0	2	2	3	3	3	4	80	5	100
Sh-RPS3	0	0	0	0	1	1	1	1	20	1	20

All mice were dissected at the end of the experiment in the lung metastatsis group, and the intact lungs were removed. Tumor foci were observed in the lungs of mice with metastasis (**Supplemental Figure 1B**). The number of mice with lung metastasis was counted (**Supplementary Table 1**). Compared with the control group, 5 mice (100%) all developed lung metastasis, only 1 (20%) had lung metastases after knockdown of RPS3. We further confirmed the occurrence of lung metastasis through tissue sections (**Figure 7H**).

## Discussion

Recently, some RPs have been revealed to be involved in regulating tumor functions. For example, RPS15A was found to promote the progression of gastric carcinoma by activating the Akt pathway (13), and RPL11 expression was identified as a potential biomarker for predicting 5-fluorouracil (5-FU) sensitivity (14). In the previous study of our team, it was found that vitamin D supplementation may regulated the poor prognosis of OSCC through RPS3 (15). Therefore, we continued to explore the role of RPS3 on ACC and found that it is also involved in cisplatin resistance of ACC. In addition, an important reason for the poor prognosis of ACC patients is that they are prone to invasion and metastasis. In this study, it was found that RPS3 also has a regulatory effect on the migration and invasion of ACC. RPS3 is a constituent protein of the 40S small subunit of the ribosome, which has been reported to be a putative marker of malignancy of colon cancer, osteosarcoma, hepatic cell carcinoma, glioblastoma (16–19), and mediate Chemotherapy resistance in gastric cancer (20).

In this study, we found that high RPS3 expression in 73 ACC patient samples was associated with both lymph node metastasis, lung metastasis, and poor prognosis. After clarifying the function of RPS3 in ACC metastasis and cisplatin resistance (**Figures 2**, **3**), we tried to explore how RPS3 exerts its regulatory function. Studies have shown that RPS3, a non-REL subunit of NF-kB, interacts with the p65 subunit through its K homology (KH) domain, resulting in NF-κB-induced transcriptional activation associated with cell survival and epithelial-stromal transformation (10). Co-IP combined with mass spectrometry was used to search for differential proteins that directly bind to RPS3. Finally, STAT1, which is the intersection of cell-cell adhesion and response to drug pathways, was selected as the research object. STAT1 is a member of the signal transducer and activator of transcription (STAT) family, primarily activated by IFN (21). In this study, it was found that the level of RPS3 had no significant effect on the mRNA and protein levels of STAT1, but was positively correlated with the phosphorylation of STAT1, while the downstream P65 activation changed accordingly. Some studies suggest that upon some stimulation, even phosphorylated dimerized STAT1 cannot enter the nucleus, but exercise specific function in the cytoplasm (22). We speculate that STAT1 can bind to RPS3 in ACC, after activation, it plays a corresponding regulatory role in the downstream NF-κB signaling pathway. Previous studies believed that the mechanisms by which RPs act as regulatory downstream factors are complex, these mechanisms may require some chaperones (23), or the presence of other factors involved in nuclear transposition (24), it also has been reported that intracytoplasmic STAT1 can also bind to NF-κB and activate downstream gene expression in a STAT3-like manner (25, 26), those studies are consistent with our speculation. Of course, further experiments are needed to clarify this conclusion. In further experiments, we applied inhibitors and activators of NF-κB to ACC cells, and clarified the effect of RPS3/STAT1/NF-κB pathway activation on ACC migration, invasion and cisplatin resistance.

*In vivo* experiment, 2 mice in the control group started to have grasping weakness 10 days after tumor formation, which is also in line with the characteristics of nerve infiltration and metastasis in the early stage of ACC (27). The results showed that the dysfunction rate was significantly reduced after RPS3 knockdown. It is thought that knockdown of RPS3 reduces the invasive ability of ACC, and on the other hand, it may inhibit tumor growth and reduce nerve compression.

## Conclusion

RPS3 is highly expressed in ACC patients and is associated with the prognosis and survival of ACC patients. The RPS3/STAT1/NF-kB pathway may play an important regulatory role in ACC migration, invasion and chemoresistance (**Figure 7**). ACC with high expression of RPS3 may have more neural invasion and lung metastasis, and knockdown of RPS3 may be an effective means to inhibit the development of ACC. As a new therapeutic target of ACC, its clinical application value is worthy of attention and further exploration.

## Data Availability Statement

The original contributions presented in the study are included in the article/**Supplementary Material**. Further inquiries can be directed to the corresponding authors.

## Ethics Statement 

The animal study was reviewed and approved by Sun Yat-Sen University Animal Care and Use Committee 202200086.

## Author Contributions

ZQH: Conceptualization, methodology. XR: Data curation, writing- original draft preparation. ZXH: Visualization, investigation. YW: Validation, data curation. RC, YC: Writing- reviewing and editing. All authors contributed to the article and approved the submitted version.

## Funding

This work was supported by grants from Guangzhou Science and Technology Project (#202103000093), Science and Technology Program of Guangdong (#2020A1515111069, #2021A1515111121), China Postdoctoral Science Foundation (#2021M693619), Science Foundation for Young Scholars of Sun Yat-sen Memorial Hospital (YXQH202013). The Key Laboratory of Malignant Tumor Gene Regulation and Target Therapy of Guangdong Higher Education Institutes, Sun-Yat-Sen University (Grant KLB09001), Key Laboratory of Malignant Tumor Molecular Mechanism and Translational Medicine of Guangzhou Bureau of Science and Information Technology [(2013)163].

## Conflict of Interest

This research was conducted in the absence of any commercial or financial relationships that could be construed as a potential conflict of interest.

## Publisher’s Note

All claims expressed in this article are solely those of the authors and do not necessarily represent those of their affiliated organizations, or those of the publisher, the editors and the reviewers. Any product that may be evaluated in this article, or claim that may be made by its manufacturer, is not guaranteed or endorsed by the publisher.

## Supplementary Material

Supplementary Figure 1 Knockdown of RPS3 inhibited ACC invasion of the sciatic nerve and lung metastasis in nude mouse model. **(A)** Dissection of the hindlimb on the tumor-bearing side of the mouse. The sciatic nerve in the RPS3 knockout group was the same as that in the blank group; the sciatic nerve in the control group was wrapped and squeezed by the tumor. **(B)** The lung tissue samples of nude mice in the control group and RPS3 knockdown group. The tumors was observed in the control group, and the lung tissue without tumor in the knockdown group.

Supplementaty Table 1 The occurrence of lung metastases after RPS3 knockdown.

Supplementary Video 1 Blank group mouse model sticker experiment.

Supplementary Video 2 Sh-RPS3 group mouse model sticker experiment.

Supplementary Video 3 Control group mouse model sticker experiment.
