# Supplementary figures and images for: RPS3 Promotes the Metastasis and Cisplatin Resistance of Adenoid Cystic Carcinoma
Source: Front Oncol. 2022 Jun 30;12:804439. doi: 10.3389/fonc.2022.804439 (PMC9280127; doi:10.3389/fonc.2022.804439)

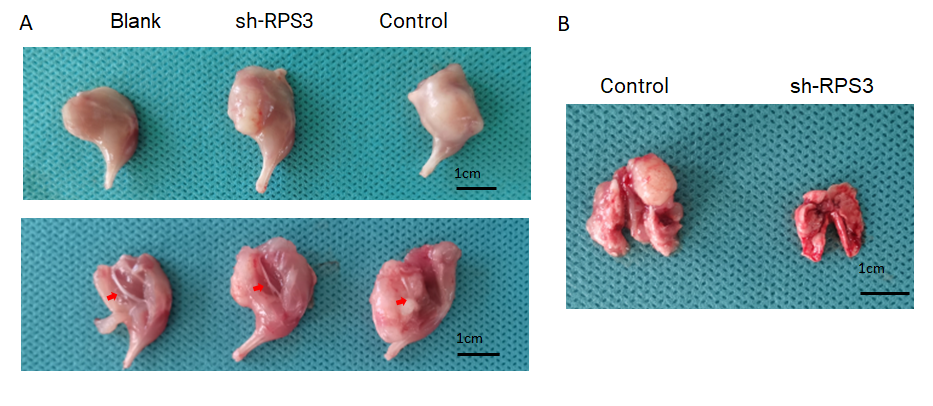

Supplement: Supplementary Figure 1 — Knockdown of RPS3 inhibited ACC invasion of the sciatic nerve and lung metastasis in nude mouse model. (A) Dissection of the hindlimb on the tumor-bearing side of the mouse. The sciatic nerve in the RPS3 knockout group was the same as that in the blank group; the sciatic nerve in the control group was wrapped and squeezed by the tumor. (B) The lung tissue samples of nude mice in the control group and RPS3 knockdown group. The tumors was observed in the control group, and the lung tissue without tumor in the knockdown group. [file Image_1.tif]
