# Supplementary material for: RPS3 Promotes the Metastasis and Cisplatin Resistance of Adenoid Cystic Carcinoma
Source: Front Oncol. 2022 Jun 30;12:804439. doi: 10.3389/fonc.2022.804439 (PMC9280127; doi:10.3389/fonc.2022.804439)
Supplement: Supplementaty Table 1 — The occurrence of lung metastases after RPS3 knockdown. [file Table_1.docx]

Supplemental Table 1. The occurrence of lung metastases after knockdown of RPS3

|  | Lung Metastases | Non-Lung Metastases | Lung Metastases Rate (%) |
| --- | --- | --- | --- |
| Control | 5 | 0 | 100 |
| Sh-RPS3 | 1 | 4 | 20 |
